# Intraoperative Fluoroscopy for Minimally Invasive Periacetabular Osteotomy: A Standardized Step‐By‐Step Operative Technique

**DOI:** 10.1111/os.70426

**Published:** 2026-09-04

**Authors:** Matthias Wittauer, Batool Bosakhar, Jitendra Balakumar

**Affiliations:** ^1^ Melbourne Orthopaedic Group Melbourne Australia; ^2^ University of Basel Basel Switzerland

**Keywords:** fluoroscopy, hip dysplasia, minimally invasive surgical procedures, osteotomy

## Abstract

**Background:**

Periacetabular osteotomy (PAO) is an established joint‐preserving procedure for symptomatic acetabular dysplasia and other forms of acetabular malalignment. Although minimally invasive techniques have reduced surgical morbidity while maintaining correctional accuracy, the procedure remains technically demanding and relies heavily on intraoperative fluoroscopic guidance. Existing descriptions of minimally invasive PAO provide limited detail regarding the systematic use of fluoroscopy throughout the osteotomy sequence. This article describes a standardized fluoroscopy‐guided technique for minimally invasive PAO.

**Operative Technique:**

The procedure is performed through an 8–10 cm inguinal incision with preservation of key soft‐tissue structures. A sequential series of fluoroscopically guided osteotomies of the superior pubic ramus, ischium, supra‐acetabular region, and posterior column are performed using defined radiographic landmarks and checkpoints. Biplanar fluoroscopy is integrated throughout the procedure to confirm osteotome trajectory, osteotomy depth, fragment mobility, and final acetabular correction. Intraoperative assessment includes restoration of acetabular coverage, version, and inclination, with confirmation of lateral center‐edge angle, Tönnis angle, anterior wall index, and posterior wall index. Technical pearls, pitfalls, and strategies to minimize neurovascular injury are highlighted.

**Conclusion:**

This standardized fluoroscopic protocol provides a reproducible framework for minimally invasive PAO. By integrating step‐specific radiographic guidance with key anatomical landmarks, the technique aims to improve osteotomy control, optimize acetabular correction, and enhance procedural safety, particularly during the learning phase of this complex hip‐preserving procedure.

## Graphical abstract

Standardized intraoperative fluoroscopy provides a reproducible step‐by‐step framework for minimally invasive periacetabular osteotomy, improving the accuracy of pelvic orientation and acetabular reorientation. This technique facilitates a consistent and teachable workflow, supporting safer execution during a procedure known for its steep learning curve.

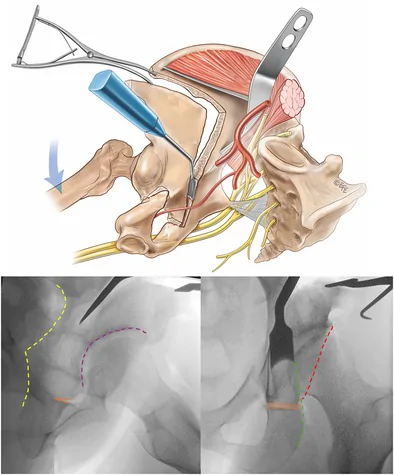

## 1 Introduction

Periacetabular osteotomy (PAO) has become a well‐established surgical treatment for developmental dysplasia of the hip (DDH) since its original description by Reinhold Ganz [1]. Over the past decades, the procedure has been widely adopted worldwide, demonstrating reliable correction of acetabular malorientation and satisfactory long‐term survivorship of the native hip [2, 3]. In young active patients, PAO has been associated with higher postoperative activity levels compared with total hip arthroplasty, without an increase in complication rates [4]. With expanding indications—including acetabular retroversion [5], borderline dysplasia [6, 7], and its successful application in selected patients with an open triradiate cartilage [8]—PAO has evolved into a gold‐standard procedure for the management of symptomatic acetabular malalignment across a broad patient population. Long‐term prospective evidence continues to support this role. A recent > 7‐year cohort demonstrated durable radiographic correction, sustained functional improvement, and a THA conversion rate of only 5.6%, reinforcing PAO's value as a joint‐preserving procedure when accurate correction is achieved [9].

Originally performed through an extensile open approach, technical refinements and growing surgical experience have enabled the development of less invasive techniques that maintain accurate radiographic correction and favorable clinical outcomes, while reducing complication rates [10]. While previous reports have described minimally invasive PAO techniques from an anatomic perspective, they lack clear guidance on the use of intraoperative fluoroscopy, which is essential for the successful and safe completion of the procedure [11, 12].

The aim of this article is to provide a comprehensive, step‐by‐step description of intraoperative fluoroscopy for minimally invasive periacetabular osteotomy. Detailed anatomical illustrations and adjunctive radiographic guidance are included to support the safe, accurate, and reproducible execution of one of the most technically demanding procedures in hip‐preserving surgery.

## 2 Operative Technique

### 2.1 Patient Selection

This technique is indicated for skeletally mature patients with closed triradiate cartilage and symptomatic acetabular dysplasia, defined radiographically by a lateral center‐edge angle < 20°–25° and/or a Tönnis angle > 10°. Adequate hip mobility is a prerequisite, including flexion ≥ 100°, absence of a fixed flexion contracture, and a congruent joint throughout the arc of motion, as assessed on preoperative imaging and/or examination under anesthesia. Successful fragment reorientation depends on a congruent and mobile hip joint.

Contraindications include advanced secondary osteoarthritis (Tönnis grade ≥ 2), persistent joint incongruity that does not correct on abduction and/or flexion views, active local or systemic infection, and neuromuscular hip disorders that may compromise fragment stability or postoperative rehabilitation.

### 2.2 Patient Positioning and Surgical Approach

The patient is anesthetized on a radiolucent table, and a urinary catheter is placed. The leg is free‐draped and stabilized using a movable leg holder. An inguinal incision of 8–10 cm is made parallel to the iliac crest 1–2 cm distal to the anterior superior iliac spine (ASIS) (Figure 1A). The ASIS is situated halfway between the lateral and medial edges of the wound. The tensor fascia lata (TFL) is identified by the blue fascia lateral to the ASIS, and the fascia is incised over the TFL. The TFL is retracted laterally using the passage of the finger. The lateral femoral cutaneous nerve (LFCN) can be identified over the sartorial fascia and is protected (Figure 1A).

**FIGURE 1:**
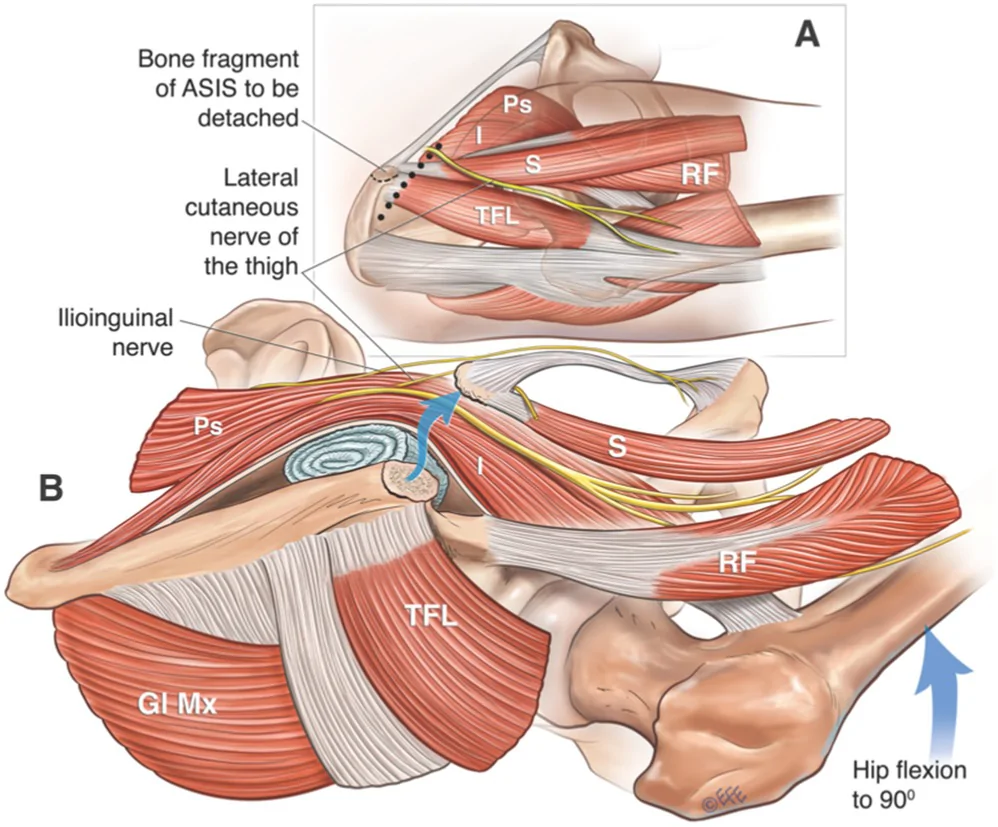
The skin incision is made parallel to the iliac crest 1–2 cm distal to the ASIS (dotted line) (A). The ASIS osteotomy with the insertion of sartorius and the inguinal ligament is retracted medially, and a gauze pack is placed in the iliac fossa subperiosteally (B). Gl Mx, gluteus maximus; I, iliacus; PS, psoas; RF, rectus femoris; S, sartorius; TFL, tensor fasciae latae.

The external oblique abdominal muscle is released from the iliac crest using cautery, taking care to stay close to bone and not to stray proximally in order to avoid injury to the ilioinguinal nerve. The periosteum is then incised along the iliac crest and continued in the plane of the TFL bed. A small osteotome is used for an ASIS osteotomy, which is left attached to the insertion of sartorius and the inguinal ligament and is retracted medially. The hip is then flexed to 90° to relax the iliopsoas. A Cobb elevator is used to subperiosteally dissect the iliacus off the inner table and down to the pelvic brim. Care is taken to descend toward the superior ramus rather than directly posterior as this may cause pressure or injury to the obturator vessels as they sit on the posterior column. A radiolucent reverse Hohmann retractor is placed on the posterior column as a lever to retract the iliopsoas medially. Care should be taken to avoid resting the retractor in the greater sciatic notch to prevent sciatic nerve injury. A gauze pack is placed in the iliac fossa using a Cobb elevator to retract the iliac muscle away from the bone (Figure 1B).

### 2.3 Osteotomy of the Pubic Ramus

The periosteum is released along the ilio‐pectineal line with cautery and a Cobb elevator. Release of this periosteum of the posterior column, especially heading toward the ramus, is crucial to release the fascia of the obturator foramen and allow inferior mobilization of the obturator neurovascular structures. A Lane‐Trethowan bone lever is placed subperiosteally anterior to the superior ramus, but medial to the iliopectineal eminence. The leg is further flexed to 80° with slight adduction to take the tension off the femoral neurovascular structures. A second bone lever is placed posterior to the superior ramus and into the obturator foramen. A straight osteotome (2 cm) is utilized for an oblique osteotomy starting at the anterior edge of the superior ramus and extending medially. This is confirmed fluoroscopically, with the cutting edge of the osteotome positioned just inferior to the superior border of the superior pubic ramus. Care is taken to remain medial to the acetabulum, thereby ensuring an extra‐articular trajectory (Figure 2).

**FIGURE 2:**
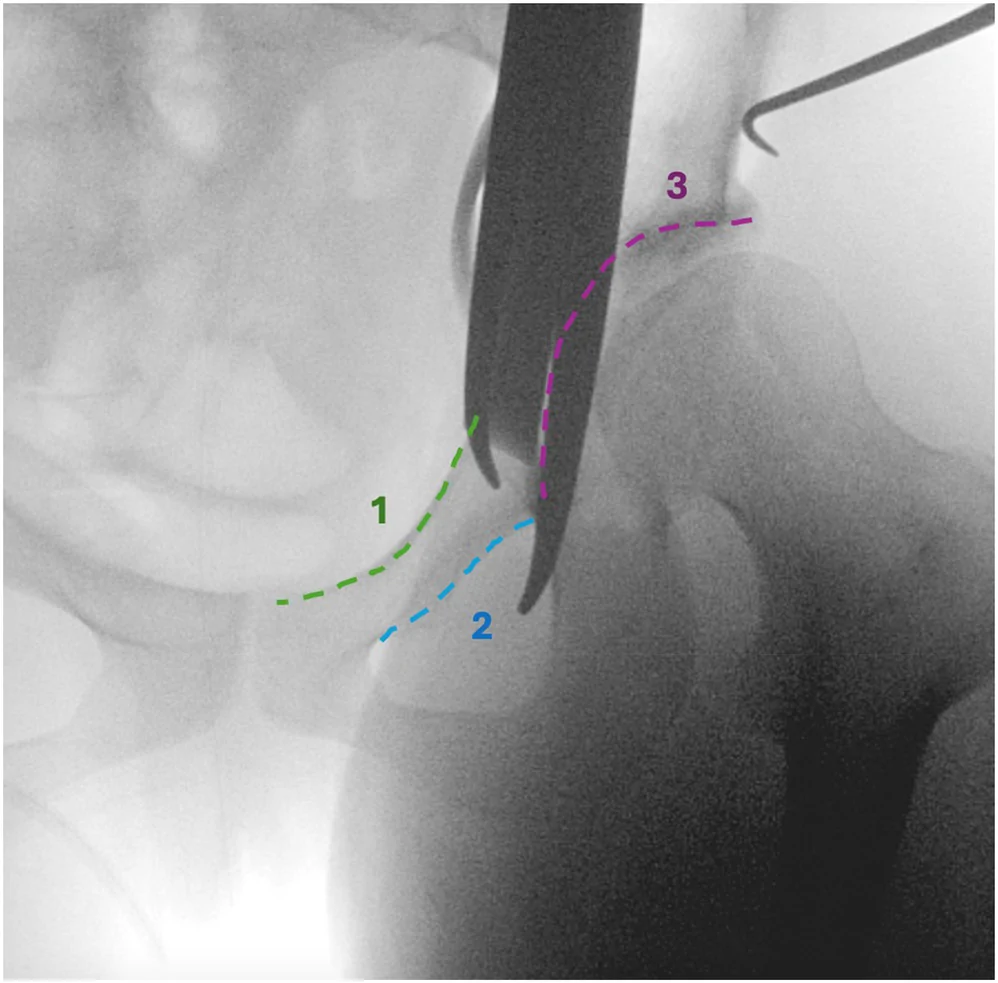
Fluoroscopic visualization of the pubic ramus osteotomy in the AP view: superior cortex of the pubic ramus (1), inferior cortex of the pubic ramus (2), and acetabulum (3).

The anterior component (outer table) is completed first to protect the obturator neurovascular bundle, which is situated posteriorly and inferiorly to the superior pubic ramus. Osteotomy is continued until a tonal change is heard, and the ramus osteotomy is completed inferiorly (Figure 3).

**FIGURE 3:**
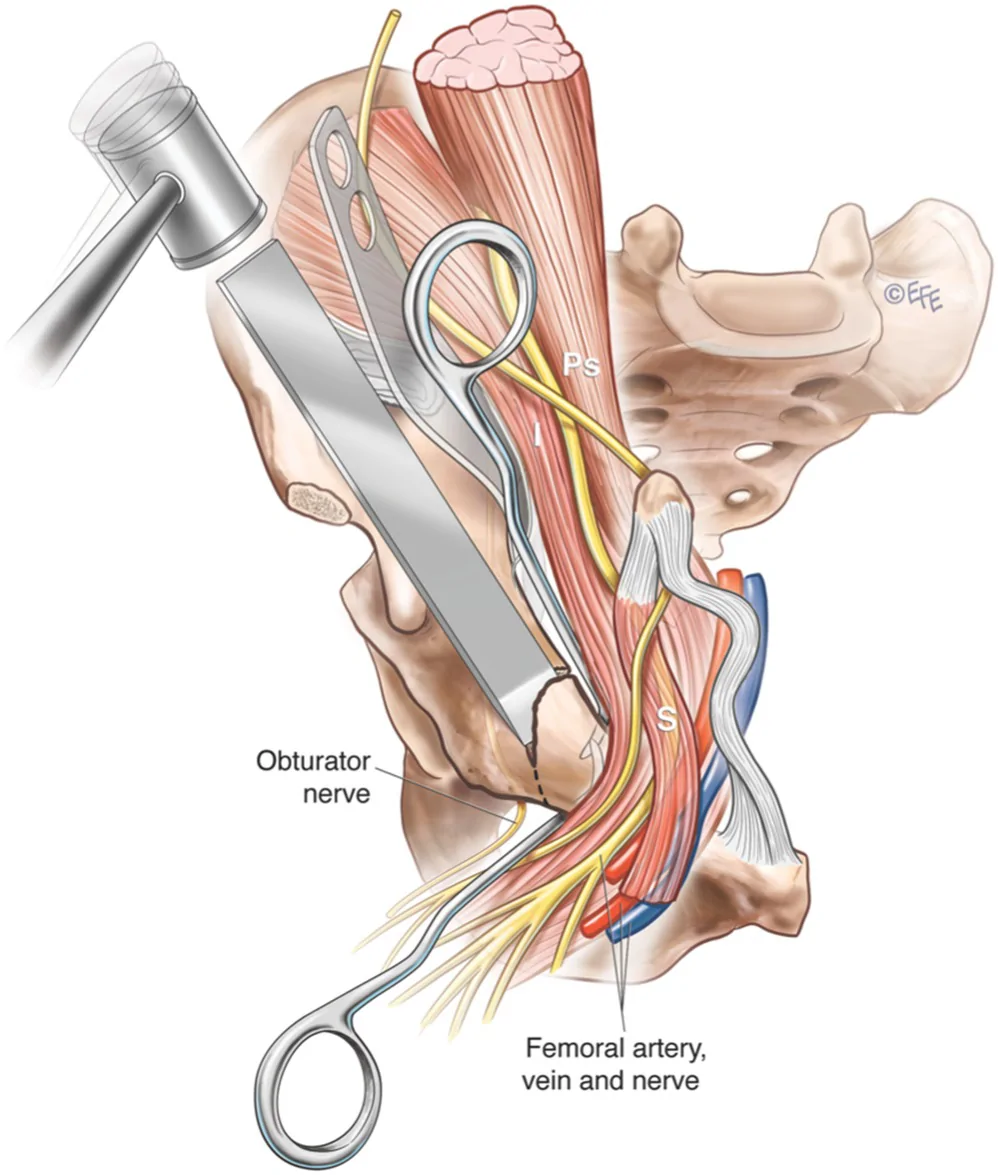
Illustration of the pubic ramus osteotomy, demonstrating the oblique cut starting at the anterior border of the superior pubic ramus and extending medially, with the neurovascular structures retracted medially. Ps, psoas; S, sartorius.

A double‐action plier is used to provide rotational force to complete the ramus osteotomy. Occasionally, the superior or inner table component of the ramus will need to be osteotomized separately with the angled osteotome. The completion of the first osteotomy can be checked with fluoroscopy and passage of a small Cobb elevator.

### 2.4 Osteotomy of the Ischium

The interval between the medial capsule and the psoas is opened utilizing a Cobb elevator, and the iliocapsularis is bluntly retracted off the capsule. A Cobb elevator is passed down to the ischium, with subperiosteal retraction of the obturator externus distally. This interval is then further opened using a large Mayo scissor with a blunt dissection technique. The psoas is retracted medially and anteriorly using a large Langenbeck retractor while the radiolucent reverse Hohmann is placed onto the ischium and used as a lever (or “laryngoscope”) to allow safe passage of the small Ganz angled osteotome to the ischium (Figure 4). The starting point of the ischial osteotomy is distal to the joint in the subcotyloid groove and curving toward the base of the ischial spine.

**FIGURE 4:**
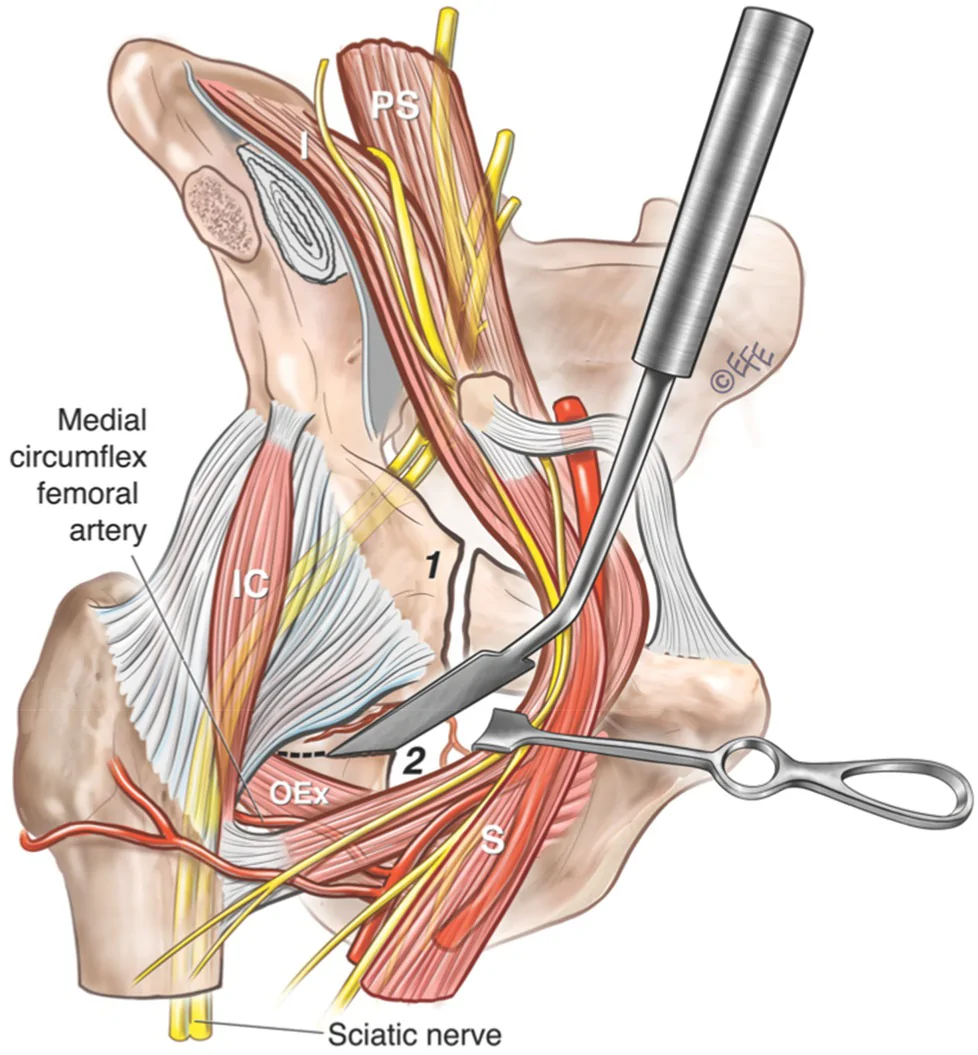
Illustration of the passage of the angled osteotome for the second osteotomy, the partial cut of the anterior ischium (2) after completion of the pubic ramus osteotomy (1). The osteotome is positioned within the interval between the anterior column posteriorly, the iliocapsularis and joint capsule laterally, and the psoas medially. The dotted line represents the starting point of the second osteotome. Note that the osteotome position depicted in this illustration does not indicate the initial direction of advancement; the osteotomy trajectory should be oriented away from the sciatic nerve. IC, iliocapsularis; OEx, obturator externus; Ps, psoas; S, sartorius.

The ischium is partially osteotomized under biplanar fluoroscopic guidance (ap and iliac oblique view) in three separate steps to a depth of 1–2 cm (Figure 5). First, the medial cortex of the ischium is osteotomized, followed by the midportion, and lastly the lateral cortex. The lateral cortex osteotomy needs to involve only the most anterior part to protect the sciatic nerve, which sits posterior to the lateral cortex of the ischium. The hip can be externally rotated during this component of the osteotomy to protect the sciatic nerve and increase the ischiofemoral space. Although the starting point of the osteotome may change on the anterior surface of the ischium, the blades of the osteotome are always pointed medially to not breach the posterolateral cortex and thus prevent sciatic nerve injury. The depth of osteotomy is confirmed on an iliac oblique (Judet) view with fluoroscopy in 50° of external rotation.

**FIGURE 5:**
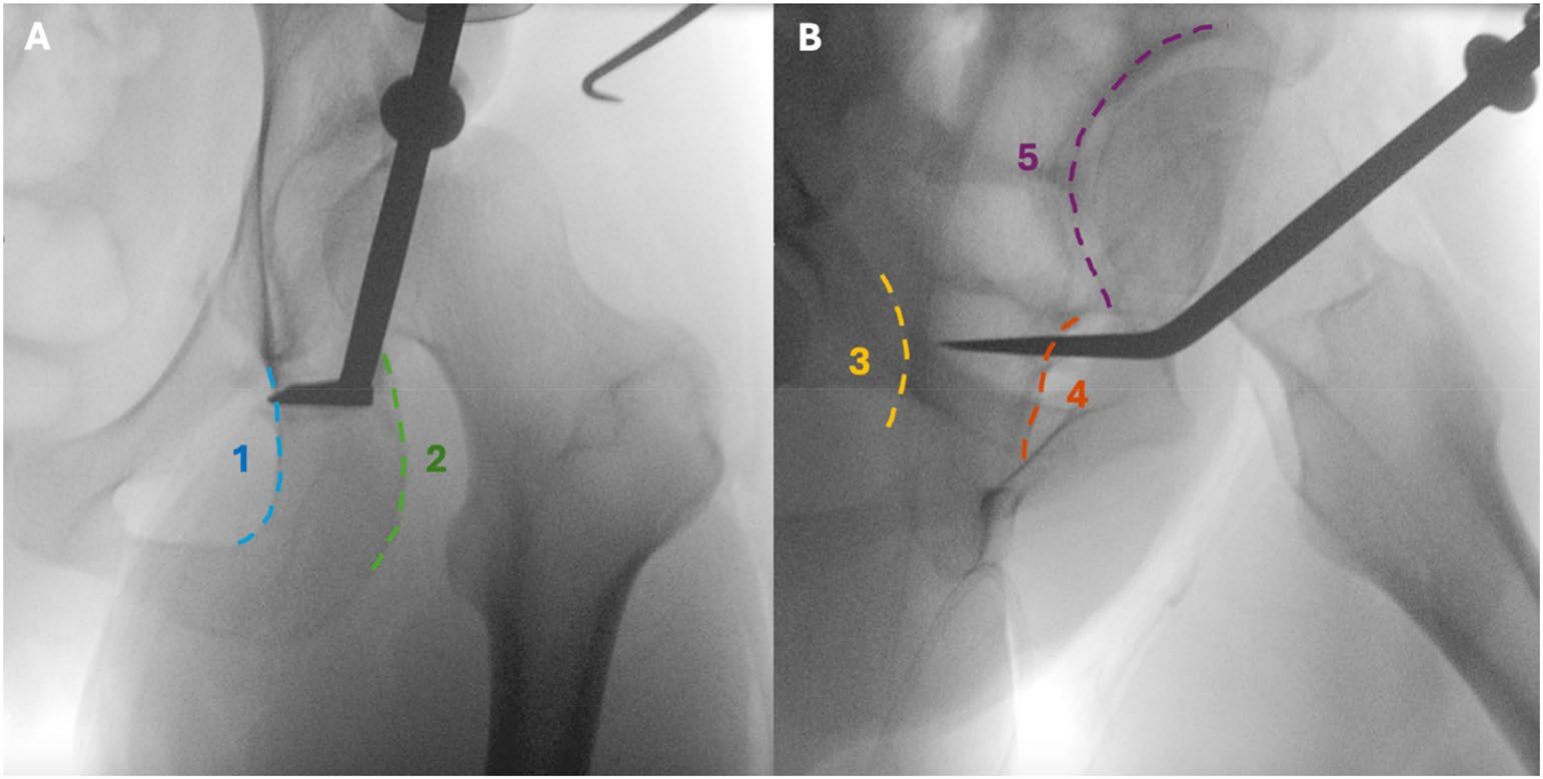
Fluoroscopic visualization of the partial ischial osteotomy in AP view (A): Medial (1) and lateral cortex (2) of the ischium; in iliac oblique view (B): posterior cortex (3) and anterior cortex (4) of the ischium. The starting point of the ischial osteotomy is distal to the joint (5) in the subcotyloid groove.

A baseline AP hip is confirmed on fluoroscopy with all instruments removed and the leg in neutral extension. The pelvic tilt is adjusted under fluoroscopy by angling toward inlet or outlet views to replicate the preoperative standing AP pelvis radiograph, with confirmation achieved by matching the appearance of the obturator foramina (Figure 6).

**FIGURE 6:**
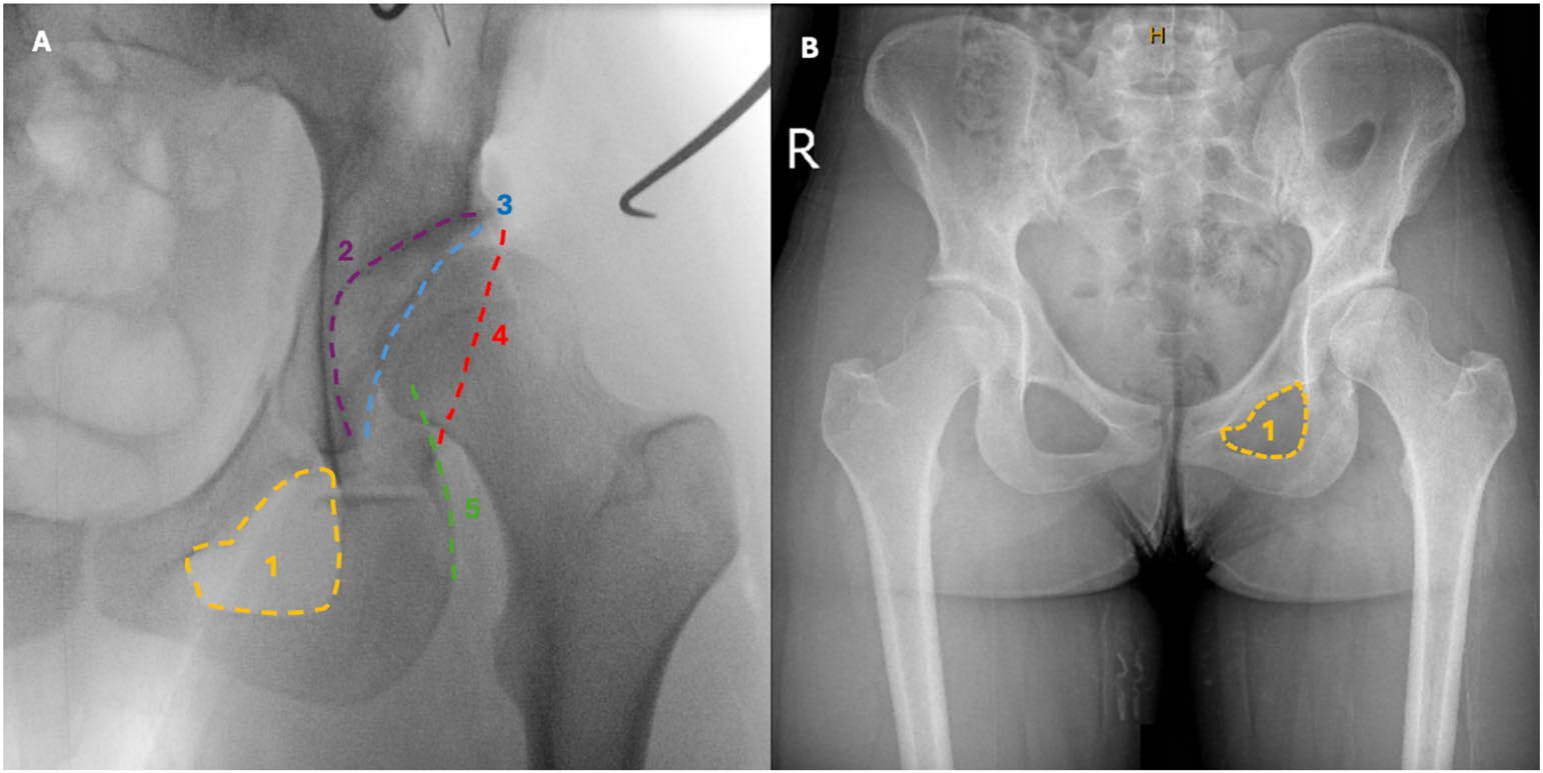
Baseline fluoroscopic image of the hip, with the intraoperative obturator foramen (A1) matching the appearance of the preoperative view (B1). Landmarks include the acetabulum (2), anterior wall (3), posterior wall (4), and the lateral cortex of the ischium (5).

### 2.5 Supra‐Acetabular Osteotomy

The supra‐acetabular cut is performed with the hip in 70° of flexion. The Howarth periosteal elevator is used for subperiosteal dissection of the outer table at the planned supra‐acetabular osteotomy site and protection of the gluteal musculature and superior gluteal neurovascular bundle. A precision oscillating saw is used to complete the supra‐acetabular cut along the planned osteotomy line. The saw blade is perpendicular to the patient rather than the pelvic wing to avoid violating the acetabulum (Figure 7).

**FIGURE 7:**
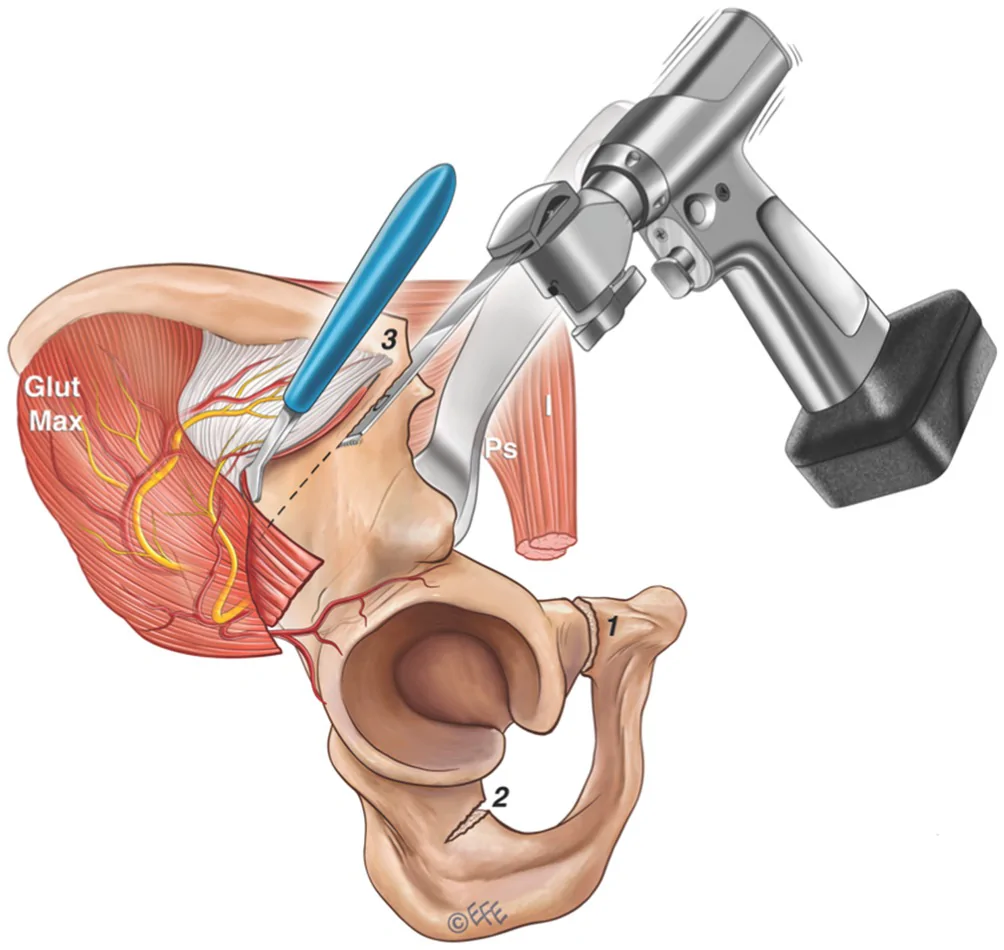
The supra‐acetabular osteotomy (3) is completed with an oscillating saw after completion of the pubic ramus osteotomy (1) and partial ischial osteotomy (2). The gluteal musculature is protected with a Howarth periosteal elevator. Glut Max, gluteus maximus; I, iliacus; Ps, psoas.

A turning point between the supracetabular and retro‐acetabular osteotomy is located 2 cm from the pelvic brim and superior to the sciatic notch to both protect the sciatic nerve and allow suitable acetabular bone stock.

### 2.6 Retro‐Acetabular Osteotomy

The retro‐acetabular osteotomy is begun on the medial side at 120° to the supracetabular osteotomy using a straight osteotome in the direct plane of the false profile. This is advanced until the point equidistant between the posterior acetabulum using a straight osteotome, and then completed along the medial cortex with the angled Ganz osteotome (Figure 8).

**FIGURE 8:**
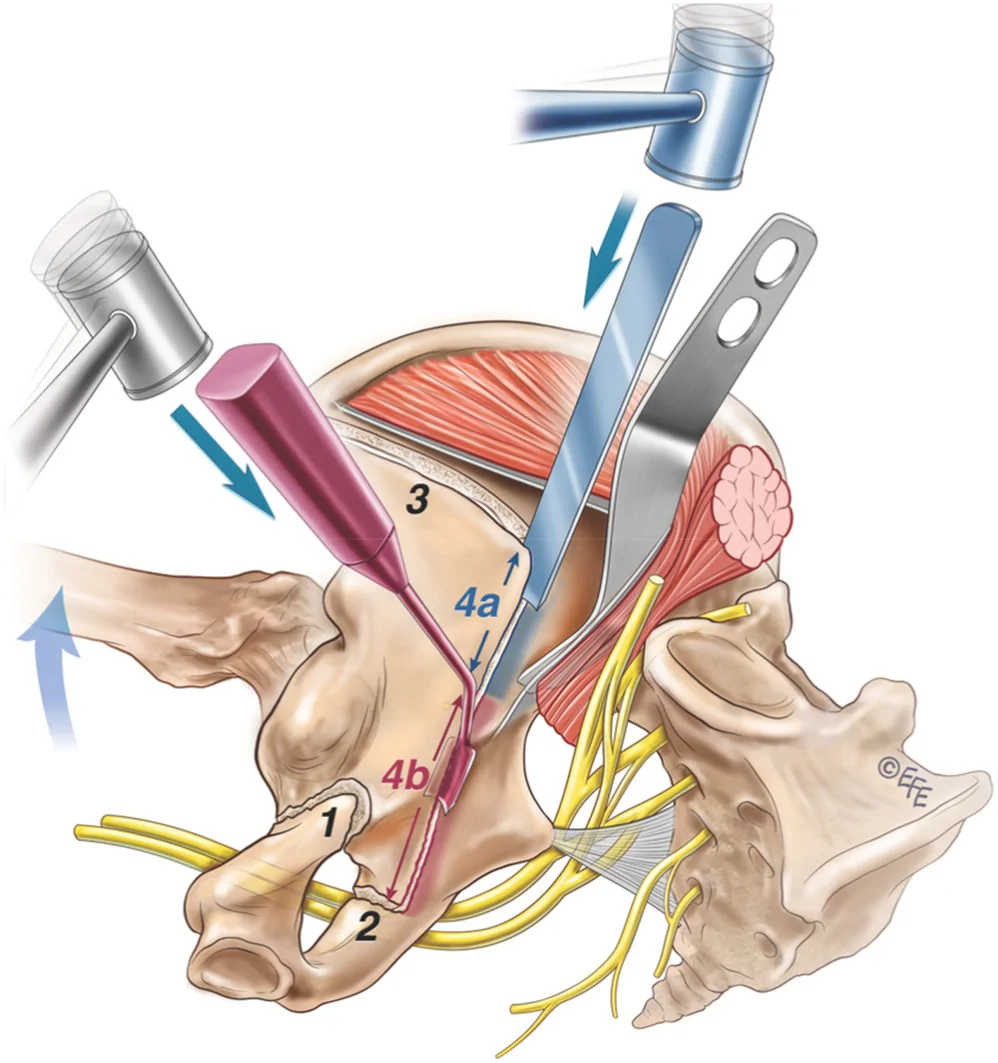
Illustration of the medial component of the retro‐acetabular osteotomy with the hip in flexion. A straight osteotome is used for the initial portion of the osteotomy (4a) until it reaches the pelvic brim, after which it is exchanged for an angled osteotome to complete the cut and connect with the partial ischial osteotomy (4b).

The corner between the supra‐acetabular and retro‐acetabular cut is opened in a medial to lateral direction with the angled osteotome. A laminar spreader is used to open the supracetabular cut, and then the angled osteotome is used to complete the lateral component of the retro‐acetabular cut (Figure 9).

**FIGURE 9:**
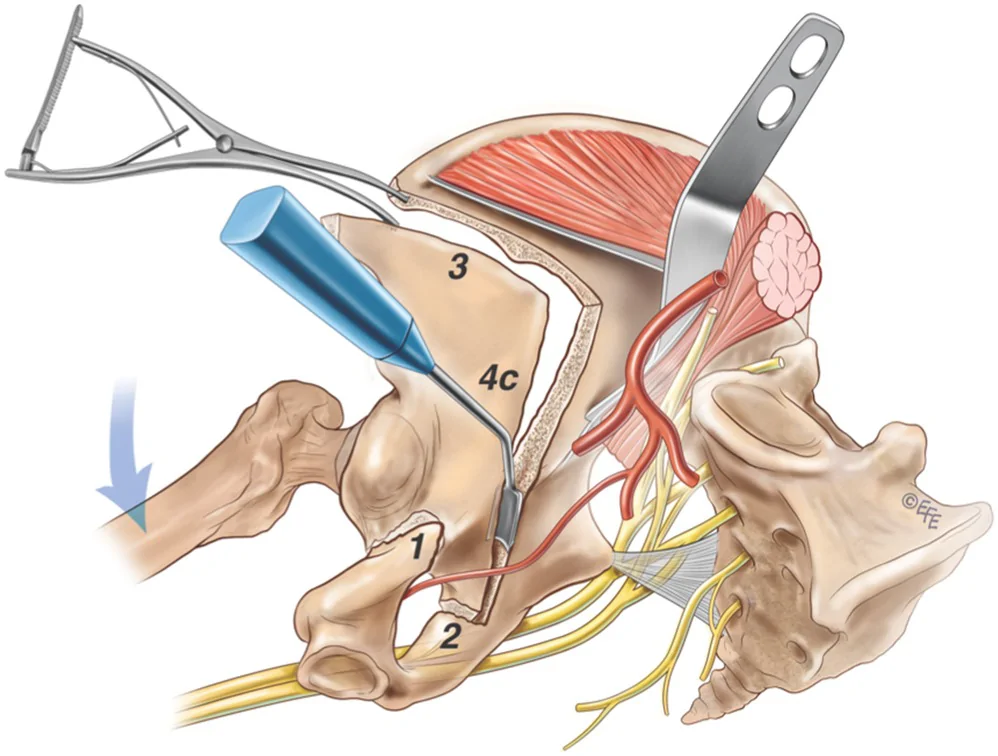
Illustration of the lateral component of the retro‐acetabular osteotomy (4c) with the hip in extension to relieve tension of the sciatic nerve.

Biplanar fluoroscopy is used to confirm the position as the osteotome advances along the lateral cortex of the posterior column. During this step, the leg is extended and abducted to increase the distance of the sciatic nerve from the osteotomy field. It is essential to use the lateral cortex of the ischium—rather than the posterior wall—as the reference for the lateral border to minimize the risk of sciatic nerve injury; the osteotome should never be advanced lateral to this landmark (Figure 10). The osteotome is then progressed further until it connects with the partial ischial osteotomy.

**FIGURE 10:**
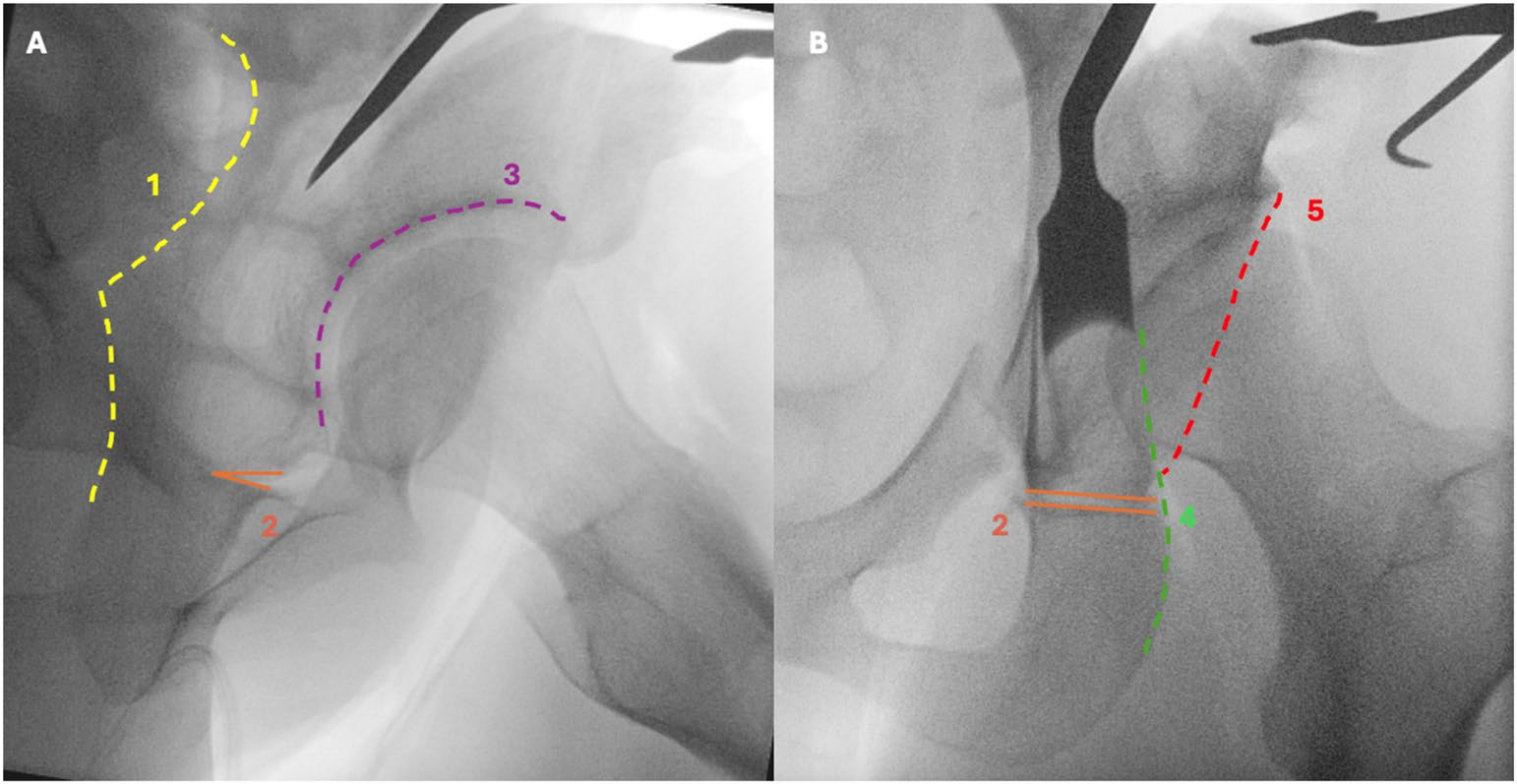
Fluoroscopic visualization of the osteotome trajectory for the retroacetabular cut in the iliac oblique view (A), passing between the sciatic notch and posterior ischial cortex (1) and the acetabulum (3), and directed toward the partial ischial osteotomy (2). In the AP view (B), the lateral cortex of the ischium (4), rather than the posterior wall (5), serves as the reference landmark; the osteotome should never be positioned lateral to this line to avoid sciatic nerve injury.

Completion of the last part of the osteotomy (inferolateral cortex of the ischium) is confirmed by circular freedom of the acetabular fragment with a 5 mm Schanz pin placed supra‐acetabular but within the acetabular fragment and a reduction forceps placed around the pubic ramus of the acetabular fragment.

### 2.7 Acetabular Correction

The acetabular fragment is then mobilized into the desired position, taking into account lateral rotation, medialization, anteversion, and acetabular flexion. Care is taken to avoid excessive medialization in patients with a narrow ischiofemoral space to prevent impingement. The correction is applied and fixed with 2.5 mm K wires placed from the iliac crest extending into the acetabular fragment. The wires are placed in a fan shape for superior fixation (anterior lateral to posterior medial direction). The correction is confirmed on intra‐operative fluoroscopy, both on AP and iliaque oblique view.

The lateral center‐edge angle, Tönnis angle, anterior wall index, and posterior wall index are then confirmed in the AP view, and assessment of possible impingement is performed with the hip at 90° flexion and internal rotation in iliac oblique view (Figure 11).

**FIGURE 11:**
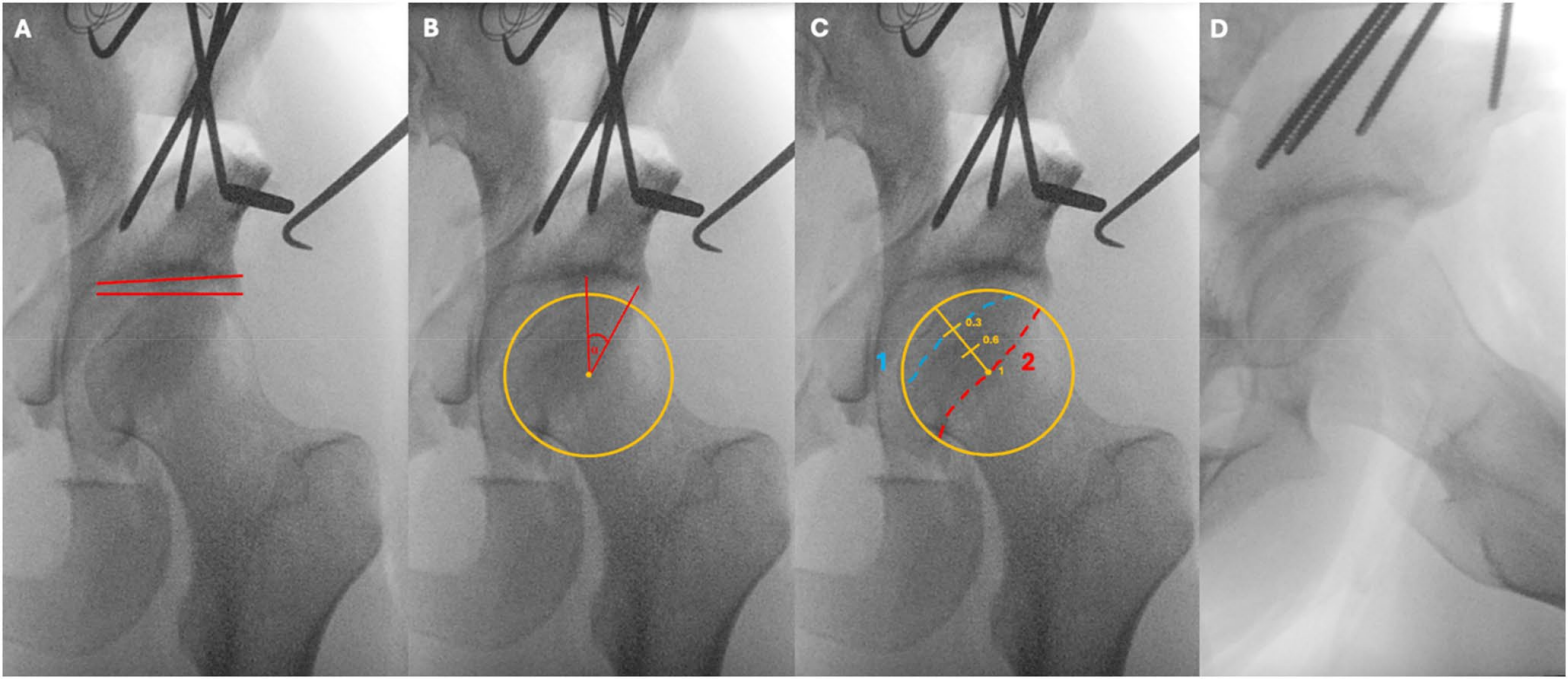
Intraoperative assessment of acetabular correction in the AP view using the Tönnis angle (A), lateral center‐edge angle (B), anterior (C1) and posterior wall indices (C2), and assessment for femoro‐acetabular impingement in the iliac oblique view (D).

The K wires are then replaced with screws in a similar fan‐shaped construct (Figure 12).

**FIGURE 12:**
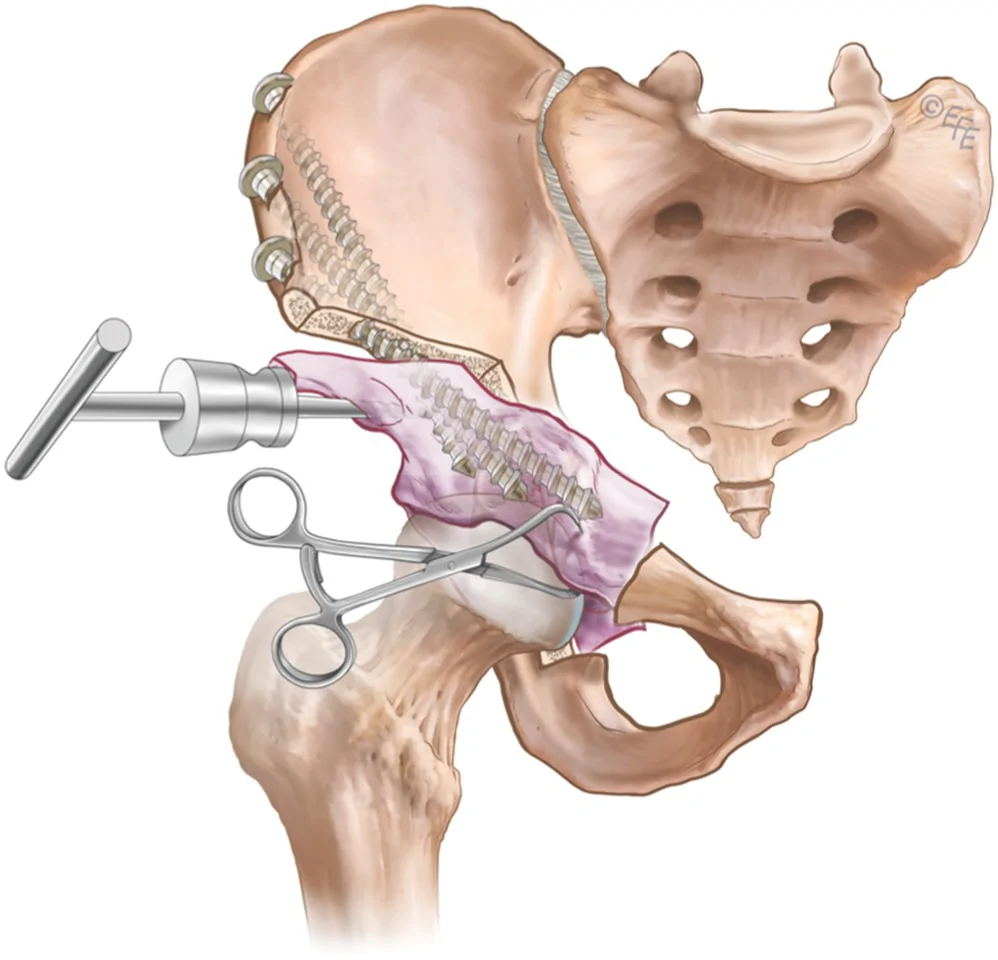
Illustration showing the mobilization of the acetabular fragment and screw fixation in fan construct to achieve internal fixation stability.

Final correction of osteotomy is recorded. Hemostasis is achieved, local anesthetic is administered, and the osteotomy site is bone grafted with resection of protruding bone from the acetabular fragment and synthetic bone graft. The anterior superior iliac spine osteotomy is repaired using transosseous sutures to the iliac crest. The fascial layers over the iliac crest and over the TFL are closed, taking care to avoid the LFCN. The wound is closed in layers, and dressings are placed. Postoperative radiographs confirm the achieved correction and appropriate screw placement (Figure 13).

**FIGURE 13:**
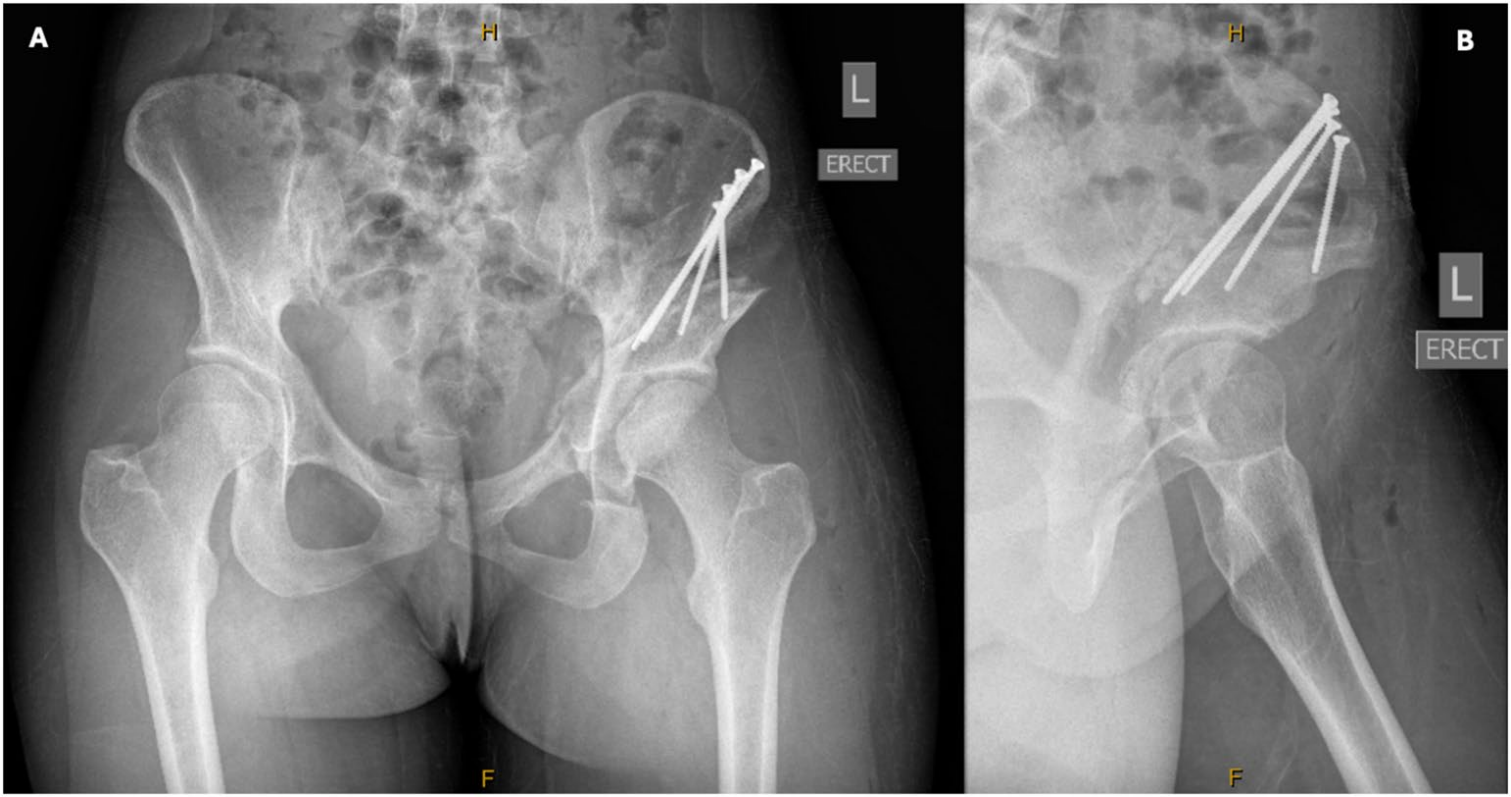
Postoperative radiographs demonstrating the achieved correction and appropriate screw placement following PAO in AP (A) and iliac oblique (B) views.

Table 1 summarizes the key technical pearls, potential pitfalls, and relevant fluoroscopic landmarks.

**TABLE 1:** Overview of technical pearls, pitfalls, and intraoperative fluoroscopy guidance.

Step	Technical pearl	Pitfall	Intraoperative fluoroscopy guidance
ASIS osteotomy	Stay lateral on TFL to define interval	Injury to LFCN	Guided by macroscopic anatomy (Figure 1A)
External oblique abdominal muscle release from iliac crest	Stay close to iliac bone and do not stray proximally	Injury to ilioinguinal nerve	Guided by macroscopic anatomy (Figure 1B)
Placement of medial retractor	The reverse Hohmann retractor is placed on the posterior column as a lever to retract the iliopsoas medially with the hip in flexion	Placing the retractor in the greater sciatic notch causing sciatic nerve injury	Guided by macroscopic anatomy
		Performing this step with the hip in extension may result in traction injury to the femoral nerve	
Pubic ramus osteotomy	Complete cut of anterior/superior cortex first Osteotomy placed medial to hip joint	Injury to obturator neurovascular bundle Intraarticular cut	AP pelvis (Figure 2) with Visualization of the superior and inferior cortex of the pubic ramus Medial positioning of the osteotome relative to the acetabulum
Ischial osteotomy	Medially directed osteotome for cut of lateral cortex away from sciatic nerve Control of depth of partial osteotomy of ischium (confirmed on iliac oblique Judet view)	Sciatic nerve injury with osteotome going laterally Completion posteriorly of the ischial cut	AP pelvis (Figure 5) with identification of medial and lateral cortex of the ischium Iliac oblique view (Figure 5) with visualization of anterior and posterior cortex of the ischium
Supraacetabular cut	Protection of gluteal musculature and superior gluteal neurovascular bundle with a Howarth periosteal elevator during the cut	Injury to gluteal musculature and superior gluteal neurovascular bundle	Guided by macroscopic anatomy
Retroacetabular cut *medial cortex*	Controlled cut of medial cortex along the posterior column between acetabulum and sciatic notch	Intra‐articular (too anterior) or posterior column (too posterior) fracture	Iliac oblique view (Figure 10 left) with visualization of the posterior cortex of the posterior column and acetabulum
Retroacetabular cut *central portion/lateral cortex*	Controlled central cut with hip in more extension and adduction	Passage of osteotome laterally to lateral cortex of ischium leading to sciatic nerve injury	AP pelvis (Figure 10 right) with visualization of the lateral cortex of the ischium and posterior wall
		High risk when using the posterior wall as a border for osteotome positioning	
Acetabular correction	Adjusting the ap pelvis image to the pre‐operative standing AP X‐ray taking into account pelvic tilt and rotation.	Under−/overcorrection due to failed adjustment/correction for pelvic tilt	AP pelvis with matching appearance of obturator foramina (Figure 6)
	Correcting the position of the acetabulum to achieve optimal coverage, with the following target parameters: LCEA = 30°–40°, Tönnis angle = 0°–5°, AWI = 0.3–0.51, and PWI = 0.81–1.14.		Measurement of hip angles/indices in AP view and assessment for femoroacetabular impingement in iliac oblique view with flexion and internal rotation of hip (Figure 11)
Acetabular correction *medialization*	Avoid medialization in patients with narrow ischiofemoral space	Creation of ischiofemoral impingement	AP pelvis

Abbreviations: AP, anteroposterior; ASIS, anterior superior iliac spine; AWI, anterior wall index; LCEA, lateral center‐edge angle; LFCN, lateral femoral cutaneous nerve; PWI, posterior wall index; TFL, tensor fascia lata.

Table 2 provides step‐specific guidance for each osteotomy, including termination markers and recommended management strategies for technical deviations.

**TABLE 2:** Step‐specific osteotomy guidance, termination markers, and management of technical deviations.

Osteotomy step	Start point/direction	Depth/termination marker	Management of technical deviations
Pubic ramus	Superior cortex of superior ramus, oblique cut extending medially	Tonal/pitch change on mallet strike signals cortical breach; confirmed on AP fluoroscopy against superior/inferior ramus cortices	If osteotome trends posterior/inferior toward obturator vessels, withdraw, re‐confirm position on AP fluoroscopy, and re‐direct anteriorly before re‐advancing
Ischium (partial)	Distal to the joint in the subcotyloid groove, curving toward the base of the ischial spine; medial cortex first, then midportion, then lateral cortex (with osteotome directed medially)	Depth limited to 1–2 cm; lateral cortex cut kept to the most anterior portion only with osteotome directed medially; confirmed on iliac oblique (Judet) view at 50° external rotation	If the osteotome trends posterolaterally toward the sciatic nerve, stop, externally rotate the hip to increase the ischiofemoral space available for the osteotome, and reconfirm the osteotome position of the lateral cortex on AP fluoroscopy before proceeding.
Supra‐acetabular	Along the planned line marked after subperiosteal exposure with the Howarth elevator; saw blade kept perpendicular to the floor, not the pelvic wing	Cut proceeds to a turning point 2 cm from the pelvic brim and superior to the sciatic notch	If the trajectory angles toward the acetabular dome, reposition the saw perpendicular to the floor (not the iliac wing) and recheck the turning‐point landmark before continuing
Retro‐acetabular — medial component	Begins medially at 120° to the supra‐acetabular cut, in the plane of the false profile; straight osteotome first, exchanged for angled osteotome at the pelvic brim	Advanced to the point equidistant between the posterior acetabular wall and the partial ischial osteotomy	If the cut trends intra‐articular (too anterior) or into the posterior column (too posterior), withdraw and reorient using the false‐profile plane on fluoroscopy before readvancing
Retro‐acetabular—central/lateral component	Corner between supra‐ and retro‐acetabular cuts opened medial‐to‐lateral with a laminar spreader, then completed with the angled osteotome	Lateral cortex of the ischium (not the posterior wall) is the non‐crossing boundary; hip extended and abducted during this step to protect the sciatic nerve	If the osteotome passes lateral to the ischial cortex landmark, stop immediately, reconfirm on AP fluoroscopy, and withdraw to the ischial‐cortex reference before resuming
Acetabular fragment mobilization	Correction applied via Schanz pin and reduction forceps once circumferential fragment freedom is confirmed	Target LCEA 30°–40°, Tönnis angle 0°–5°, AWI 0.30–0.51, PWI 0.81–1.14, confirmed on AP and iliac oblique fluoroscopy with obturator‐foramen matching to the preoperative film	If under‐ or overcorrection is seen, release provisional K‐wires, remobilize the fragment, and re‐image before definitive fixation; avoid medialization in patients with a narrow ischiofemoral space to prevent iatrogenic impingement

Abbreviations: AP, anterior–posterior; AWI, anterior wall index; LCEA, lateral center‐edge angle; PWI, posterior wall index.

## 3 Discussion

PAO remains the cornerstone joint‐preserving surgical procedure for symptomatic acetabular dysplasia in skeletally mature patients, following its introduction by Ganz and colleagues in 1988 [1, 3, 13]. The defining biomechanical advantage of the Bernese technique, namely simultaneous multiplanar acetabular reorientation while preserving posterior column continuity, provided superior primary fragment stability and facilitated rehabilitation compared with earlier osteotomy techniques, which either required violation of the posterior column or imposed prolonged post‐operative immobilization, limiting their clinical applicability in young active patients. The original series, however, also documented the considerable technical demands of the procedure in its earliest form, including operative times exceeding 5 h, mean intraoperative blood loss approaching 3000 mL, and a notable risk of intra‐articular osteotomy propagation [13]. Compared with the original extensile approach, the present technique utilizes a limited 8–10 cm inguinal incision with clearly defined fluoroscopic checkpoints at each stage of the osteotomy while preserving both the fundamental osteotomy geometry described by Ganz and the integrity of the posterior column [1, 14, 15, 16]. This evolution mirrors the broader trajectory of PAO refinement over the subsequent four decades, during which technical innovations, progressive reduction in incision length, soft‐tissue interval preservation, and the systematic incorporation of intraoperative imaging have improved the procedure's safety profile without compromising correctional accuracy or long‐term survivorship [12].

### 3.1 Comparison With Previously Described Minimally Invasive PAO Techniques

This minimally invasive technique emphasizes a structured, reproducible surgical workflow combined with technical modifications intended to improve safety and intraoperative control. Particular emphasis is placed on sequential intraoperative radiographic guidance. Although previous reports of minimally invasive PAO have described anatomical landmarks and soft‐tissue interval development in detail, they have largely lacked a systematic account of intraoperative fluoroscopy [11, 12]. Intraoperative imaging is indispensable for the safe and accurate execution of each osteotomy step, particularly when direct visualization is constrained by the limited surgical corridor. The present description addresses this gap by integrating step‐specific fluoroscopic checkpoints into a coherent, reproducible protocol supported by anatomical illustrations and representative intraoperative images at each critical stage [14, 17, 18]. Appendix A provides an overview of the key differences between the presented technique and the two most frequently cited minimally invasive PAO technical notes.

### 3.2 Value of Intraoperative Fluoroscopy

The reliability of intraoperative fluoroscopy as a surrogate for postoperative radiographic assessment in PAO has been validated in the literature. Several studies demonstrated excellent to good intraclass correlation coefficients between fluoroscopic and postoperative standing radiographic measurements for the lateral center‐edge angle, Tönnis angle, anterior wall index, and posterior wall index [14, 19, 20]. Accurate adjustment of pelvic tilt and rotation by reproducing the obturator foraminal profile of the preoperative standing AP pelvis forms the foundation of reliable intraoperative fluoroscopic assessment [14, 21] and is therefore established as a mandatory initial step prior to the osteotomy sequence. Building on this framework, fluoroscopy is then used dynamically throughout the procedure to guide each corrective step in real time. Intraoperative fluoroscopy additionally avoids logistical complexity and increased radiation exposure associated with formal intraoperative plain radiography [18, 21], making it a more practical and safer real‐time imaging modality for PAO. Verification confirms achieved correction against a known target, whereas real‐time guidance actively directs osteotome trajectory as the procedure unfolds. Emerging CT‐based and computer‐assisted navigation platforms offer an alternative real‐time modality; systematic review data show comparable operative times and reduced radiation exposure versus conventional fluoroscopy, but at the cost of preoperative CT imaging, added equipment, and expense that limit widespread adoption [17, 22]. Fluoroscopy therefore remains the more accessible real‐time tool for most units performing minimally invasive PAO.

### 3.3 Learning Curve for Performing PAO

A structured stepwise technique may help address the well‐established steep learning curve associated with PAO by providing a consistent and reproducible workflow. Haertle et al., using cumulative summation analysis in 118 consecutive PAOs, identified a case‐volume inflection point of 26 procedures before operative duration plateaued and concluded that a minimum training exposure of 50 PAOs per surgeon per year at a high‐volume center is required to attain consistent proficiency [23]. Novais and colleagues further reported that despite formal fellowship training, major complications did not decrease significantly between a surgeon's first and subsequent 20 PAOs, highlighting that even structured training carries residual risk during early independent practice [24]. A structured‐mentorship program for a geographically isolated low‐volume surgeon demonstrated that good radiographic outcomes and low complication rates are achievable when technical guidance is formalized and consistently applied, a principle that written fluoroscopic protocols can meaningfully support, particularly in units where high‐volume PAO training is inaccessible [25]. Notably, junior surgeons have been shown to use significantly greater fluoroscopy time during PAO than their more experienced counterparts, and this increased reliance on imaging during the learning phase underscores the value of a structured fluoroscopic protocol to guide decision‐making in real time [26, 27]. This reflects that fluoroscopy is essential when visualization is limited, but without a structured approach, more imaging alone does not improve decision‐making. A recent systematic review and meta‐analysis pooling early versus late–experience PAO cohorts found that operative time decreased significantly with surgeon experience, whereas complication rates did not differ significantly between phases, suggesting that efficiency gains are more consistently demonstrable than complications reduction alone [28].

### 3.4 Neurovascular Complications Associated With PAO

Neurovascular complications may plausibly be reduced by careful, anatomically guided control of osteotome positioning; however, the available literature does not establish that the specific fluoroscopic workflow described in this article results in superior clinical outcomes. In a multicenter series of 1760 PAOs, Sierra et al. reported a 2.1% incidence of major femoral or sciatic nerve injury, with complete neurologic recovery occurring in only half of affected patients [29]. More contemporary minimally invasive experience supports lower incidence, in a single‐surgeon series of 715 minimally invasive PAOs, no permanent major nerve injury occurred, although symptomatic LFCN dysesthesia requiring nerve neurolysis was still seen in a small subset, reflecting this nerve's persistent vulnerability despite technical refinement [30]. The present technique incorporates several measures intended to address recognized mechanisms of nerve injury. During the ischial osteotomy, the lateral cortex of the ischium, rather than the posterior wall, is used as the lateral boundary on AP fluoroscopy to help limit excessive posterolateral osteotome propagation, a recognized mechanism of sciatic nerve injury. Similarly, extension and abduction of the leg during the central portion of the retro‐acetabular osteotomy are intended to increase the distance between the osteotomy trajectory and the sciatic nerve. Furthermore, the LFCN deserves acknowledgement as an underappreciated source of postoperative morbidity. Prospective nerve‐conduction studies have documented LFCN injury rates approaching 90% following PAO, with approximately two‐thirds of patients remaining symptomatic at 3 years [31]. The deliberate identification and protection of the LFCN over the sartorial fascia prior to fascial incision is therefore a step of genuine clinical consequence rather than merely surgical courtesy. These technical measures are based on established anatomical considerations and surgical experience, but their effectiveness in reducing nerve injury remains to be formally evaluated.

Optimal acetabular correction, targeting a lateral center‐edge angle of 30°–40° [32] an acetabular index of 3°–13° [33] an anterior wall index of 0.30–0.51, and a posterior wall index of 0.81–1.14 [34], is informed by biomechanical and survivorship evidence demonstrating that both under‐ and overcorrection compromise native hip longevity [32, 34]. These factors require an extremely detailed intraoperative assessment using fluoroscopy, where poor fragment placement because of inadequate coverage laterally or too much anterosuperior rotation will dictate future joint loads and survival without undergoing further arthroplasty procedures. Specifically, the lack of anterior wall reconstruction index has been identified as an independent risk factor for conversion to THA surgery at follow‐up [34]. This relationship has recently been corroborated prospectively; both THA conversions in a > 7‐year cohort occurred in hips with intraoperative LCEA correction of less than 12°, underscoring that precision of correction, rather than surgical approach alone, is a key determinant of long‐term survivorship [9]. Overcorrection of the anterior center‐edge angle and acetabular roof is independently associated with postoperative reduction in hip flexion and internal rotation [35], and may generate anterior femoroacetabular impingement, a complication first described by Myers and Ganz after PAO [36]. Intraoperative assessment of hip range of motion after correction, as incorporated into this protocol, provides a real‐time safeguard against this specific failure mode [30].

### 3.5 Limitations

This article has several limitations. Most importantly, it is a technical description intended to provide surgeons with clear and standardized guidance, rather than a clinical outcomes study. Accordingly, no case series, complication rates, or other clinical outcomes are reported, and the proposed fluoroscopic checkpoints have not yet been validated in a consecutive patient cohort. Furthermore, the technique reflects the experience of a single surgeon and may therefore be influenced by the associated learning curve and reliance on fluoroscopic expertise. Although the correlation between fluoroscopic and postoperative radiographic measurements has been independently validated [14, 19, 20], several of the specific checkpoints described are based on the authors' surgical experience rather than a formal consensus process, as no consensus statement defining intraoperative fluoroscopic checkpoints for this procedure currently exists in the literature. Prospective evaluation in a consecutive clinical cohort, including operative time, fluoroscopy time, radiographic correction, clinical outcomes, and complications, is needed to establish the safety, reproducibility, and clinical effectiveness of the described technique.

## 4 Conclusion

This article presents a standardized, step‐by‐step fluoroscopic guide designed to facilitate the safe, accurate, and reproducible execution of PAO through a limited surgical approach. By integrating clearly defined intraoperative fluoroscopic checkpoints with detailed anatomical guidance, the described technique aims to improve osteotomy control, optimize acetabular correction, and minimize complications, particularly during the learning phase of this complex procedure. Although further clinical outcome studies are required, this structured fluoroscopic workflow may serve as a practical adjunct for surgeons performing minimally invasive PAO and support wider reproducibility of the technique.

## Author Contributions

**Matthias Wittauer:** conceptualization, methodology, investigation, resources, writing – original draft, writing – review and editing. **Batool Bosakhar:** conceptualization, investigation, writing – original draft, writing – review and editing, visualization. **Jitendra Balakumar:** conceptualization, resources, writing – review and editing, project administration, supervision.

## Funding

The authors have nothing to report.

## Disclosure

All authors listed meet the authorship criteria according to the latest guidelines of the International Committee of Medical Journal Editors (ICMJE). All authors have made substantial contributions to the conception and design, drafting or critical revision of the manuscript, and approve the final version for submission. All authors agree with the content of the manuscript and consent to its submission for publication.

## Ethics Statement

All intraoperative and radiographic images are de‐identified. The patient depicted provided specific written informed consent for publication of these images, separate from consent for the surgical procedure. Institutional review board approval was not required, as this manuscript describes a technical surgical technique using de‐identified images from a single patient and does not constitute a human‐subjects outcomes study; this determination is consistent with our institution's policy for technique‐description publications.

## Conflicts of Interest

J.B. reports royalties from Medacta and Arthrex, consultancy for Stryker, and membership of an advisory board for Medacta. The other authors declare no conflicts of interest.

## Data Availability

The data that support the findings of this study are available from the corresponding author upon reasonable request.

## Appendix A Comparison of the Presented Technique With Previously Described Minimally Invasive PAO Techniques.

**Table:**

Domain	Present technique	Khan et al. [11]	Thiagarajah et al. [12]
Surgical approach	8–10 cm inguinal incision parallel to iliac crest, modified Smith‐Petersen interval, ASIS osteotomy	Modified Smith‐Petersen approach	Minimally invasive anterior approach with described soft‐tissue interval preservation
Osteotomy sequence	Pubic ramus → ischium (partial) → supra‐acetabular → retro‐acetabular (medial then central/lateral)	Sequence described anatomically; imaging checkpoints not systematically specified per step	Sequence described with an emphasis on minimizing intraoperative risk; imaging checkpoints not systematically specified per step
Fluoroscopic views used	Step‐specific biplanar protocol: AP + iliac oblique (Judet) at defined checkpoints for every osteotomy, plus obturator‐foramen matching for pelvic tilt	Fluoroscopy referenced as an adjunct; view‐by‐view protocol not detailed	Fluoroscopy referenced as an adjunct; view‐by‐view protocol not detailed
Protected structures	LFCN, ilioinguinal nerve, obturator neurovascular bundle, sciatic nerve (lateral ischial cortex as explicit non‐crossing boundary), femoral neurovascular structures	Standard anatomic protection described qualitatively	Explicit focus on minimizing neurovascular risk, described qualitatively
Assessment of final correction	Intraoperative fluoroscopic LCEA, Tönnis angle, AWI, PWI, and impingement testing at 90° flexion/internal rotation, referenced against preoperative standing AP pelvis	Correction assessed on fluoroscopy; specific indices not systematically itemized	Correction assessed on fluoroscopy; specific indices not systematically itemized

Abbreviations: AP, anteroposterior; ASIS, anterior superior iliac spine; AWI, anterior wall index; LCEA, lateral center‐edge angle; LFCN, lateral femoral cutaneous nerve; PWI, posterior wall index.
